# Diseases of the Kidneys

**Published:** 1881-03

**Authors:** 


HALL'S

### JOURNAL OF HEALTH

AND

MISCELLANY.

E. H. GIBBS, A. M., M. D. - - EDITOR.

Founded in 1854, and published Monthly without Interruption since that Time.

### DISEASES OF THE KIDNEYS.

DISEASES of the kidneys are of far less frequency than is generally supposed.

There is a very general belief that any illness that is accompanied with irregularity of the secretions from the kidneys is ample evidence of disease of those organs.

When, however, we understand that the fluid portions of the waste matters from the body are mainly passed out of the system through the urinary passages, and that the quantity and character of these effete matters depend on, or are influenced by, a variety of circumstances, not inconsistent with perfect health, we shall find little cause for anxiety, on account of the varied and peculiar appearances of the discharges.

Not only is the quantity and the appearance of these secretions affected by what we eat and drink, and by extremes of heat and cold, but even more by mental impressions. Any nervous excitement, as fear, anxiety, grief, or anger, frequently causes an entire suppression of these secretions for hours.

There is a popular dread of what is commonly called "kidney complaint," and when a nervous man is suffering from any ailment that he cannot define; if he can locate a dull pain in the region of the kidneys, and if he notices that the urine is highly colored, and that there is a sediment on the bottom of the vessel in the morning, his worst fears are confirmed and he is ready to invest in any, or all, of the advertised specifics for kidney diseases. It is not to be wondered at that venders of quack medicines of this sort can procure any number of genuine certificates from well-known and reputable persons, who feel certain that they have been saved from untimely death by these wonderful preparations.

I believe that most physicians will agree with me in the opinion that not more than one person in five who seeks medical advice, under the impression that he has kidney disease, has any serious trouble of that sort. Such persons are usually dyspeptics, and treatment appropriate for that disease usually cures the kidney trouble. But kidney diseases are not infrequent, and when neglected are likely to have a painful and fatal termination. I should, therefore, regret to have any well-grounded fears set at rest by anything I have written on this subject. It is the duty of every person to promptly seek the best medical advice in all cases of doubt. There are to-day but few diseases that at the commencement will not yield promptly to proper treatment. Bright's disease, so often fatal when once fairly established, is usually curable in the first stages. That eminent physician, Prof. Geo. B. Wood, in the second volume of his "Treatise on the Practice of Medicine," page 515, says :

"The opinion appears to have been entertained by some that this disease is very formidable ; that, when fully formed, it is in fact incurable. Though the symptoms arising from exacerbations may subside, though the various secondary affections may be for a time removed, yet the fundamental evil, it is thought, still remains, ready to be called into activity by the slightest causes, and, though the patient may live many years, he is yet always in uncertain health, and never safe. But I am convinced that this is a very erroneous view."

I am well acquainted with two gentlemen in New York, both of whom seemed to be in the advanced stages of the disease ten years ago, but who recovered, and have since seemed to be in almost perfect health, and I believe there are thousands of such cases.

Diabetes is a far more formidable disease, and more insidious in its approaches than Bright's disease. It is, therefore, more likely to become established before it is recognized.

Finally, it is safe to say that "false alarms" in regard to nearly all diseases generally proceed from derangement of the digestive organs, and the better plan to avoid them is to regulate the diet to meet the requirements of the system, which will depend on the amount of mental and bodily labor to be performed.

---

Let him who regrets the loss of time make proper use of that which is to come in the future.

An American lawyer is now attorney general of the Sandwich Islands. If in two years he doesn't own the entire country and hold the king's note for a large sum, he is no credit to the American bar.

